# Case Report: Occult mantle cell lymphoma in a synchronous rectal collision tumor with adenocarcinoma: retrospective reclassification and multispecimen molecular profiling

**DOI:** 10.3389/fonc.2026.1871711

**Published:** 2026-08-20

**Authors:** Qing Zhang, Yingjie Xiu, Junhong Li, Xin Cao

**Affiliations:** 1 Department of Pathology, Affiliated Hospital of Nantong University, Nantong, Jiangsu, China; 2 Department of Pathology, The First Affiliated Hospital of Shenzhen University, Shenzhen Second People’s Hospital, Shenzhen, China; 3 Department of Special Examination Center, Affiliated Hospital of Nantong University, Nantong, Jiangsu, China; 4 Department of Hematology, Affiliated Hospital of Nantong University, Nantong, Jiangsu, China

**Keywords:** case report, collision tumor, mantle cell lymphoma, rectal adenocarcinoma, TP53

## Abstract

Collision tumors composed of colorectal adenocarcinoma and mantle cell lymphoma are exceptionally rare, and component-resolved molecular characterization is largely lacking. We report a 65-year-old woman who initially presented with rectal adenocarcinoma and underwent radical resection followed by adjuvant capecitabine. Seven months later, she developed generalized lymphadenopathy, and excisional cervical lymph node biopsy confirmed mantle cell lymphoma with Cyclin D1 and SOX11 positivity, t(11;14)(q13;q32) rearrangement, and a Ki-67 proliferation index of approximately 40%. Retrospective review of the original rectal resection specimen identified an occult mantle cell lymphoma component at the tumor margin and within the submucosa, leading to reclassification as a synchronous rectal collision tumor composed of adenocarcinoma and mantle cell lymphoma. Targeted sequencing across four specimens revealed a shared TP53 exon 7 frameshift in the archival composite rectal specimen, the rectal lymphoma component, and the adenocarcinoma component, whereas the nodal lymphoma specimen showed a dominant NOTCH2-truncating profile without a reported pathogenic TP53 alteration. The adenocarcinoma component additionally harbored a truncating APC alteration consistent with colorectal tumorigenesis. These findings support partial molecular overlap together with compartment- and site-specific divergence, while not establishing a common clonal origin. The patient received bendamustine plus rituximab followed by zanubrutinib maintenance and remained in remission at the last follow-up. This case highlights a clinically important diagnostic pitfall, shows how retrospective pathologic review can uncover occult gastrointestinal mantle cell lymphoma in a colorectal resection specimen, and provides multi-specimen molecular evidence of spatial heterogeneity across synchronous bowel and nodal disease.

## Introduction

Collision tumors are uncommon lesions in which two histologically distinct neoplasms arise in the same anatomic site without histologic admixture. Synchronous or collision combinations of a solid tumor and lymphoma are rare overall. In the largest systematic review to date, which compiled 308 published cases across different organs and tumor types, the gastrointestinal tract was the most frequent site, adenocarcinoma was the most common solid-tumor component, and diffuse large B-cell lymphoma (21.7%) and mucosa-associated lymphoid tissue lymphoma (20.4%) were the most frequent lymphoma subtypes (1). Within this broader group, colorectal adenocarcinoma associated with mantle cell lymphoma (MCL) appears to be exceptionally rare. Isolated reports have described synchronous rectal adenocarcinoma with intestinal MCL (2) as well as earlier colonic adenocarcinoma associated with intestinal or colonic MCL (3, 4).

This setting is diagnostically challenging because MCL has a recognized propensity for gastrointestinal involvement, yet intestinal disease may be clinically unsuspected and morphologically subtle. In particular, intestinal MCL may be recognized at an early or incidental stage, sometimes alongside an advanced colonic adenocarcinoma and before the characteristic multiple lymphomatous polyposis has developed (3). Romaguera et al. subsequently noted that the reported frequency of gastrointestinal involvement in MCL had likely been underestimated and showed that many patients had microscopic disease even in macroscopically normal mucosa (5). Salar et al. further demonstrated that the great majority of newly diagnosed MCL cases in their prospective endoscopic-pathologic series had upper or lower gastrointestinal infiltration, often in the form of minute lymphoid infiltrates (6). Accordingly, when colorectal adenocarcinoma dominates a surgical specimen, a focal MCL component may be overlooked or interpreted as background lymphoid infiltration until systemic lymphoma becomes clinically apparent.

MCL is a biologically heterogeneous mature B-cell neoplasm, and TP53 disruption is now recognized as one of its most important adverse-risk features (7). However, previously reported synchronous colorectal adenocarcinoma–MCL cases have remained largely descriptive and have lacked component-resolved molecular characterization. The present case differs in three respects: the MCL component was occult within the original rectal resection specimen and was recognized only after the subsequent emergence of systemic disease prompted retrospective review; four-specimen targeted sequencing enabled component- and site-resolved molecular comparison; and the clinical course could be followed longitudinally through BR induction and zanubrutinib maintenance in a non-transplant setting. We therefore present this case not only as a rare synchronous rectal collision tumor, but also as a diagnostically deceptive and molecularly annotated presentation with extended follow-up.

## Case description

### Initial presentation and management of rectal adenocarcinoma

A 65-year-old woman presented in November 2020 with altered bowel habits and hematochezia. Colonoscopic biopsy showed moderately differentiated rectal adenocarcinoma. Contrast-enhanced computed tomography demonstrated irregular thickening and enhancement of the rectal wall, with several adjacent small lymph nodes. She subsequently underwent laparoscopic radical resection of rectal carcinoma on November 30, 2020.

**Figure 1:**
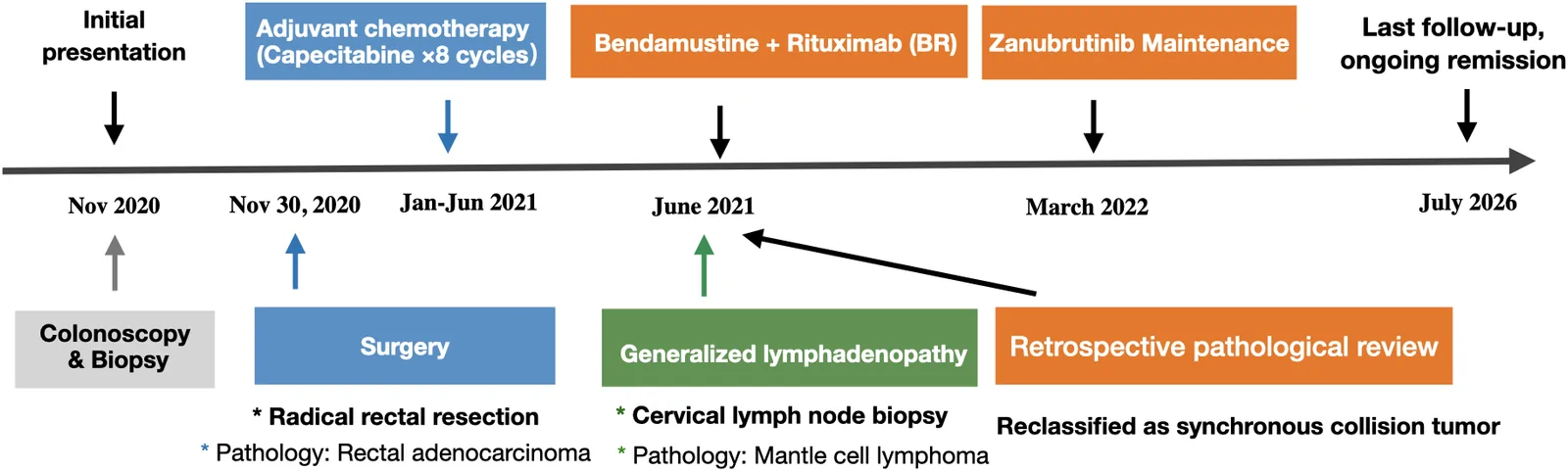
Clinical timeline of the patient with synchronous rectal adenocarcinoma and mantle cell lymphoma. The patient initially presented in November 2020 and underwent colonoscopy with biopsy, followed by radical rectal resection, with the original pathologic diagnosis of rectal adenocarcinoma. Adjuvant capecitabine was administered for 8 cycles starting in January 2021. In June 2021, the patient developed generalized lymphadenopathy, and cervical lymph node biopsy confirmed mantle cell lymphoma. During lymphoma-directed treatment, subsequent retrospective review of the original rectal resection specimen identified an occult mantle cell lymphoma component, leading to reclassification of the lesion as a synchronous rectal collision tumor. The patient then received bendamustine plus rituximab, followed by zanubrutinib maintenance from March 2022, and remained in remission at the last follow-up in July 2026. BR, bendamustine plus rituximab.

Histopathologic examination confirmed an ulcerative moderately differentiated rectal adenocarcinoma with retained mismatch repair protein expression. The tumor invaded the muscularis propria and was associated with perineural invasion. No metastatic carcinoma was identified in the 31 regional lymph nodes examined (0/31), and no definite lymphovascular invasion was identified. The overall pathologic stage was pT2N0M0 (AJCC 8th edition, stage I). From January to June 2021, the patient received adjuvant capecitabine for 8 cycles (21-day cycles; 1.5 g twice daily on days 1–14). The overall clinical course is summarized in **Figure 1**.

### Diagnostic assessment and retrospective review of the rectal specimen

During follow-up in June 2021, the patient developed a progressively enlarging cervical mass accompanied by night sweats, without fever, chills, or significant weight loss. Positron emission tomography/computed tomography (PET/CT) revealed widespread hypermetabolic lymphadenopathy involving the bilateral cervical, peripancreatic, retroperitoneal, iliac, and left inguinal regions, with the highest SUVmax of 11 observed in the dominant peripancreatic/left para-aortic lesions on the initial staging PET/CT. Mild splenomegaly with hypermetabolic foci was also noted, and peritoneal involvement was suspected.

An excisional cervical lymph node biopsy confirmed MCL. The tumor cells were positive for Cyclin D1 and SOX11, fluorescence *in situ* hybridization (FISH) demonstrated CCND1::IGH rearrangement, and the Ki-67 proliferation index was approximately 40%. On admission to the Department of Hematology, small, firm, mobile, non-tender lymph nodes were palpable in the bilateral cervical, supraclavicular, axillary, and inguinal regions. Laboratory testing showed a white blood cell count of 4.5×10^9/L, hemoglobin of 121 g/L, platelet count of 164×10^9/L, lactate dehydrogenase of 474 U/L (reference range, 120–247 U/L), and β2-microglobulin of 2.92 μg/mL (reference range, 1.0–3.0 ug/mL). Bone marrow involvement was documented. Flow cytometry identified a clonal B-cell population with the immunophenotype CD5+CD19+CD10-CD20+CD22+CD200- and surface kappa light-chain restriction, supporting marrow infiltration by MCL.

**Figure 2:**
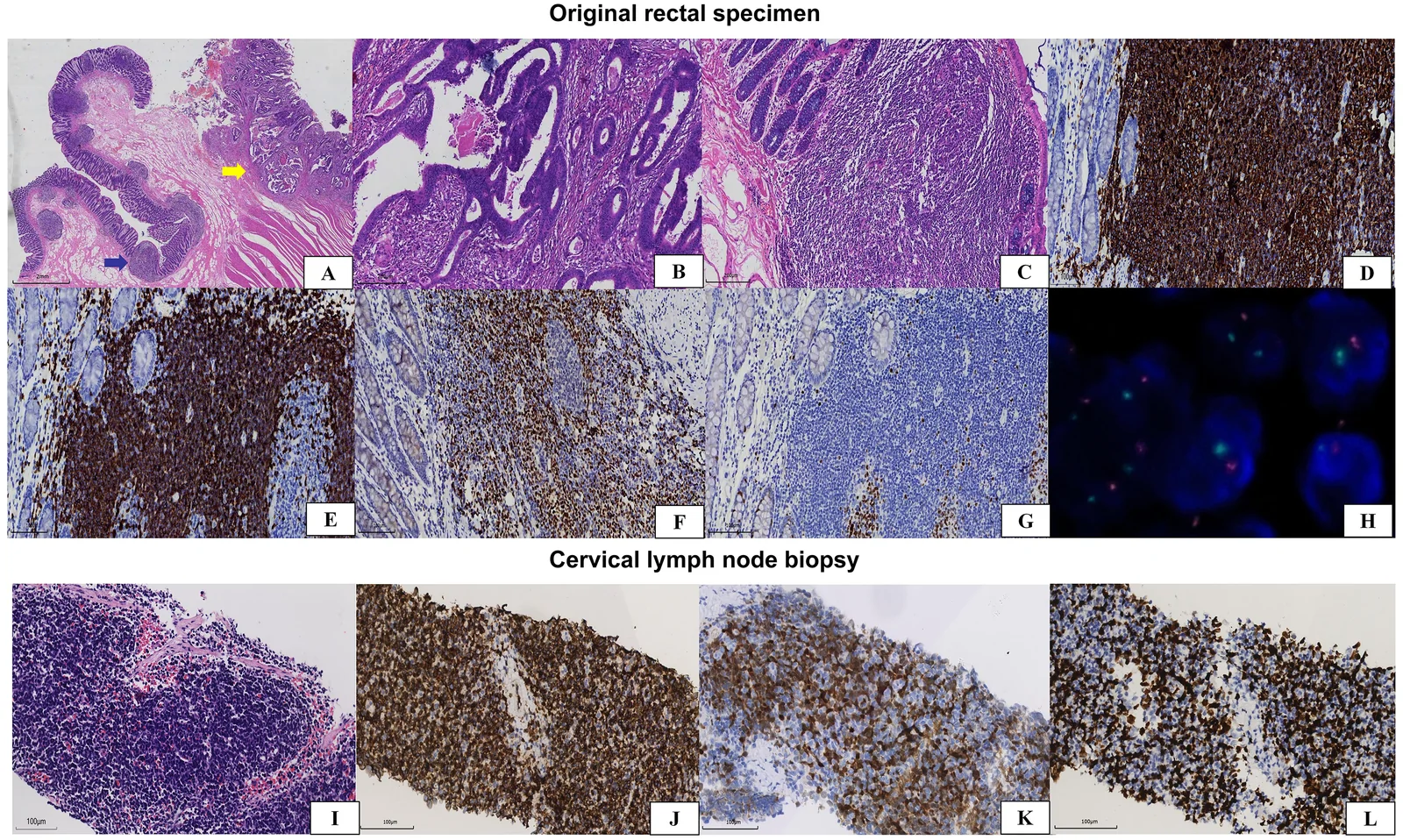
Histopathological, immunohistochemical, and FISH findings of the original rectal specimen and cervical lymph node biopsy. **(A–H)** Original rectal specimen. **(A)** H&E-stained section showing MCL tumor tissue (blue arrow) and colorectal adenocarcinoma (yellow arrow). **(B)** Higher magnification (100×) of the colorectal adenocarcinoma at the yellow arrow in **(A)**. **(C)** Higher magnification (100×) of the MCL tumor tissue at the blue arrow in **(A)**. **(D–G)** Immunohistochemical staining of the MCL region for **(D)** CD5, **(E)** SOX11, **(F)** Cyclin D1, and **(G)** Ki-67 (200×). **(H)** FISH analysis demonstrating CCND1 gene break-apart translocation (200×). **(I–L)** Cervical lymph node biopsy. **(I)** H&E-stained section (200×). **(J–L)** Immunohistochemical staining of the MCL region for **(J)** CD5, **(K)** Cyclin D1, and **(L)** Ki67 (200×).

The original colorectal biopsy and resection specimen were evaluated in the routine gastrointestinal surgical pathology workflow. After systemic MCL was diagnosed and during the subsequent lymphoma-directed treatment course, the previously resected rectal specimen was re-evaluated because the systemic lymphoma had emerged shortly after treatment of rectal adenocarcinoma. In addition to the adenocarcinoma component, sheets of small lymphoid cells that had been overlooked on the initial review were identified at the tumor margin and within the submucosa. These lymphoid cells were positive for Cyclin D1 and SOX11 and showed a low Ki-67 proliferative index of approximately 5%, supporting the presence of an occult MCL component in the original rectal specimen at the time of surgery. The archival material was technically adequate for reassessment, with preserved morphology and interpretable immunohistochemistry, FISH, and molecular testing. Representative histopathologic and immunohistochemical findings are shown in **Figure 2**. Taken together with the subsequent lymph node biopsy and bone marrow involvement, the case was reclassified as a synchronous rectal collision tumor composed of adenocarcinoma and MCL. The initial under-recognition of MCL was considered most likely related to the focal and occult distribution of the lymphoma component, its submucosal/tumor-margin location, low proliferative index, predominance of adenocarcinoma, and absence of clinical suspicion for MCL at the time of the original diagnosis.

The patient was staged as Ann Arbor stage IV, with an ECOG performance status of 2 and a MIPI score of 6 (calculated using the simplified MIPI formula (8)); with a Ki-67 proliferation index of 40% in the diagnostic cervical lymph node specimen, the MIPI-c also classified her as high risk (9). To further characterize the molecular features of the different tumor components, targeted next-generation sequencing was subsequently performed across four specimens to provide a component- and site-resolved molecular comparison. Specimen 1 represented an archival composite bowel specimen from the initial rectal resection. Specimen 2 was a cervical lymph node lymphoma specimen obtained at the time of systemic MCL diagnosis. Specimens 3 and 4 were histologically separated lymphoma and adenocarcinoma components from the original rectal resection specimen, respectively. Because the specimens differed in tissue composition and not all cross-sample comparisons were fully platform-matched, cross-sample comparison was restricted to descriptive interpretation of reported alterations; absence of a given alteration in another specimen was not taken as definitive evidence of true absence. The results are summarized in **Table 1**, and variant-level extraction from the four original reports is provided in **Supplementary Table 1**.

**Table 1:** Comparative summary of molecular findings across four specimens.

Feature	Specimen 1 Initial rectal resection specimen composite tissue	Specimen 2 Peripheral lymph node lymphoma specimen	Specimen 3 Initial rectal resection specimen lymphoma component	Specimen 4 Initial rectal resection specimen adenocarcinoma component
Panel used	Lymphoma-focused hotspot panel, 114 genes	Lymphoma-focused hotspot panel, 114 genes	MicroLym-B B-cell lymphoma panel, 254 genes	Solid tumor 120-gene tissue panel
Molecular comparison				
Reported higher-priority alterations	TP53 exon 7 c.755_780dup, p.Gly262fs, 5.89% (Level 1)	NOTCH2 exon 34 c.7198C>T, p.Arg2400*, 41.35% (Level 1) ARID1A exon 18 c.4231C>T, p.Gln1411*, 43.72% (Level 2) PCLO exon 25 c.15422C>T, p.Thr5141Met, 1.60% (Level 2)	TP53 exon 7 c.755_780dup, p.G262PfsTer92, 22.8% (IA)	TP53 exon 7 c.755_780dup, p.G262Pfs*92, 39.74% (Tier II) APC exon 6 c.642del, p.Q215Sfs*4, 15.11% (Tier II)
Additional reported alterations	ATM exon 63 p.Trp3052Cys, 3.05% ARID1A exon 18 p.Gly1665Arg, 13.44% KIR2DL1 exon 7 p.Ala275Val, 17.01% IGLL5 exon 1 p.Leu31Gln, 1.37% MYOM2 exon 5 p.Gln186His, 58.64%	ATM exon 63 p.Trp3052Cys, 54.99% MYOM2 exon 5 p.Gln186His, 46.23%	TNFRSF10B exon 4 p.G159V, 55.31% ZNF608 exon 7 p.V1405I, 51.4%	RAD51D exon 3 p.V66M, 41.50%
Key negative/ancillary findings	No NOTCH2 pathogenic variant reported	No TP53 pathogenic variant reported	No fusion gene detected IGHV result not included in this report	KRAS/NRAS/BRAF wild-type MSS; no MMR-gene variant POLE/POLD1 negative No fusion or CNV reported
Interpretive summary	Low-VAF TP53 frameshift was already detectable in the initial composite rectal specimen.	The nodal lymphoma specimen showed a dominant NOTCH2-truncating profile without a reported TP53 pathogenic variant.	Dedicated lymphoma re-testing confirmed TP53 c.755_780dup as the principal pathogenic event in the lymphoma component.	The adenocarcinoma component shared the TP53 frameshift and additionally harbored a truncating APC alteration.

CNV, copy-number variation; IGHV, immunoglobulin heavy-chain variable region; MMR, mismatch repair; MSS, microsatellite stable; VAF, variant allele frequency.

Cross-sample interpretation is descriptive because panel content, assay scope, and tissue composition differed across specimens.

### Therapeutic intervention, follow-up, and outcomes

The patient subsequently received 6 cycles of bendamustine plus rituximab (BR). After 4 cycles, PET/CT showed a partial response. At the end of induction therapy, PET/CT demonstrated persistent suspicious metabolic activity in the pelvic floor, with a Deauville score of 4. However, in conjunction with ultrasonographic findings, interval changes after anti-infective treatment, and a concurrent negative repeat bone marrow examination, multidisciplinary interpretation favored inflammatory uptake instead of active lymphoma. No confirmatory biopsy of the residual pelvic uptake was performed; therefore, the designation of complete remission reflected multidisciplinary clinical adjudication rather than strict Lugano-defined complete metabolic response (10).

Because the patient and her family declined autologous hematopoietic stem cell transplantation and rituximab maintenance, treatment was switched to zanubrutinib maintenance. Repeat PET/CT on March 20, 2024, demonstrated ongoing metabolic remission, with a Deauville score of 3. During maintenance therapy, recurrent thrombocytopenia developed, and zanubrutinib was reduced to 80 mg twice daily. At the last follow-up on July 9, 2026, the patient remained in remission for approximately 60 months from the start of lymphoma-directed treatment.

## Discussion

The value of this case lies not only in its rarity as a synchronous rectal collision tumor, but also in its clear diagnostic implication. The patient was initially managed as having isolated rectal adenocarcinoma, and the occult mantle cell lymphoma (MCL) component in the bowel specimen was recognized only after systemic lymphoma emerged and the original resection specimen was reviewed retrospectively. This sequence is clinically important because it indicates that the lymphoma component was already present at the time of surgery but was morphologically under-recognized, rather than representing a later, unrelated second malignancy. In the gastrointestinal tract, MCL may be clinically silent and histologically subtle, especially when it is early, non-polypoid, or present as a limited infiltrate adjacent to a dominant epithelial tumor. Earlier pathologic observations also showed that intestinal MCL may be detected in an early or incidental phase in association with advanced colonic adenocarcinoma, before the classic picture of multiple lymphomatous polyposis becomes evident (3). Our case therefore reinforces a practical diagnostic point for both surgical pathologists and hematopathologists: when systemic lymphoma appears soon after treatment of colorectal carcinoma, particularly in the setting of atypical lymphoid infiltrates in the original specimen, retrospective pathologic review may uncover an occult synchronous lymphoid neoplasm that was initially overlooked.

From a molecular perspective, the case is informative because it shows both overlap and divergence across anatomically and histologically distinct specimens rather than a fully uniform genomic pattern. The clearest shared event was the TP53 exon 7 frameshift, which was detectable at low variant allele frequency in the archival rectal composite specimen and was subsequently confirmed in both the rectal lymphoma component and the rectal adenocarcinoma component. By contrast, the nodal lymphoma specimen showed a dominant NOTCH2-truncating profile without a reported pathogenic TP53 alteration, whereas the adenocarcinoma component additionally harbored a pathogenic truncating APC alteration consistent with colorectal tumorigenesis. In MCL, TP53 disruption is one of the best-established adverse molecular features, whereas NOTCH pathway lesions are less frequent but have been linked to aggressive biology; genomic studies have further suggested that NOTCH2 alterations may occur as an alternative to NOTCH1 lesions in aggressive tumors (7, 11, 12). Taken together, these findings are most appropriately interpreted as showing partial molecular overlap accompanied by compartment-specific divergence, rather than a single fully shared clonal architecture. At the same time, they do not justify a definitive claim of common clonal origin, because cross-sample comparison remained constrained by differences in specimen composition and assay design, and because the archival rectal specimen was a composite sample rather than a purified single component.

The adenocarcinoma component is biologically more straightforward and helps define the interpretive boundary of this case. APC is a foundational event in colorectal tumorigenesis, whereas TP53 alteration is more commonly associated with later progression, invasive transformation, and biologic worsening (13–15). In our patient, the rectal adenocarcinoma component showed a truncating APC exon 6 alteration together with the same TP53 exon 7 frameshift detected in the rectal lymphoma component; it also showed KRAS/NRAS/BRAF wild-type status, microsatellite stability, and no mismatch repair gene alteration, fitting a conventional colorectal epithelial molecular background rather than a lymphoma-defining program. For that reason, the most defensible interpretation is not that the adenocarcinoma and lymphoma share a complete driver framework, but that a conventional colorectal cancer genomic backbone coexisted with a TP53 event that was also detected in the bowel-derived lymphoma component. In this setting, the molecular findings are best described as a shared TP53 event across selected bowel-derived components together with marked divergence between epithelial and lymphoid compartments and between bowel and nodal disease sites.

The difference between the bowel-derived lymphoma specimen and the nodal lymphoma specimen warrants particular comment, because it is central to the biologic reading of this case. One plausible explanation is spatial heterogeneity within the lymphoma itself, with different disease sites enriched for different genomic events. Another is branched evolution, in which lesions sharing only part of a molecular background subsequently diverge across anatomic compartments. To our knowledge, directly analogous case reports documenting same-patient, near-synchronous, anatomically distinct MCL lesions with discordant targeted sequencing results remain scarce. However, broader genomic studies in MCL have demonstrated subclonal heterogeneity across simultaneous or subsequent samples, including samples from different topographic sites at diagnosis. Array-CGH studies have identified multiple subclones in a substantial proportion of MCL cases (16), whole-genome and whole-exome analyses have demonstrated clonal evolution in MCL (12), and paired primary-relapse sequencing has confirmed substantial genetic heterogeneity over disease course (17). In addition, TP53 mutations in MCL may be clonal or subclonal rather than uniformly truncal events (18). Within that framework, the nodal NOTCH2-dominant profile and the bowel TP53-positive profile are not necessarily contradictory; rather, they may reflect related but non-identical subclonal representations across disease sites. This interpretation is strengthened by the fact that specimens 1 and 2 were analyzed using the same 114-gene panel and platform, reducing the likelihood that the discrepant TP53 findings are explained solely by panel mismatch. Even so, differences in specimen composition, tumor purity, and subclonal representation still preclude definitive interpretation of the TP53-negative result in specimen 2 as a biologic true negative. Although ARID1A alterations were reported in both specimens 1 and 2, these were non-identical variants—a missense variant of uncertain significance in specimen 1 and a truncating Level 2 alteration in specimen 2—and therefore should not be interpreted as a shared molecular event. By contrast, lower-priority shared variants such as MYOM2 p.Gln186His and ATM p.Trp3052Cys were observed only in specimens 1 and 2, that is, the two samples analyzed on the 114-gene hotspot platform. These remained variants of uncertain significance and were not reproduced as defining events in the component-resolved rectal lymphoma or adenocarcinoma assays. We therefore regard them as ancillary findings.

The clinical course of this patient is also notable in light of the adverse significance of TP53 aberration in MCL. Contemporary studies continue to regard TP53-mutated MCL as a biologically and clinically high-risk subgroup with inferior outcomes after conventional chemoimmunotherapy and a substantial unmet therapeutic need (7, 19). At the same time, BTK inhibition has become a central component of modern MCL management, and long-term phase 2 follow-up has shown durable activity of zanubrutinib in relapsed or refractory disease (20). The therapeutic landscape of MCL has evolved rapidly. As summarized in a recent comprehensive review, frontline management increasingly incorporates BTK inhibitors, both combined with chemoimmunotherapy and within chemotherapy-free regimens, while relapsed or refractory disease is now approached with covalent and noncovalent BTK inhibitors, CAR T-cell therapy, and bispecific antibodies (21). Against this evolving backdrop, the prolonged remission achieved in our patient with a comparatively conventional bendamustine-rituximab induction followed by zanubrutinib maintenance sequence, in a non-transplant setting, appears more favorable than might be expected. This finding should not be overinterpreted in a single case, but it does suggest that clinical behavior may not be uniform across all TP53-aberrant presentations and may also be shaped by subclonal structure, site-specific genomic composition, and subsequent BTK inhibitor exposure. More recently, Liu et al. showed that TP53-mutated MCL is itself heterogeneous and that the prognostic impact of TP53 lesions may vary by structural location (22). The TP53 p.G262PfsTer92 alteration in our case lies within the DNA-binding domain and, within that framework, may be viewed as distinct from the highest-risk hotspot category. Because this location-based framework was not developed specifically for frameshift lesions, extrapolation to TP53 p.G262PfsTer92 should be regarded as hypothesis-generating rather than definitive. More broadly, truncating or loss-of-function TP53 alterations in MCL remain associated with adverse biology overall (23). Even so, this remains an inferential interpretation in a single patient and should not be extended beyond the descriptive level.

Several limitations should be acknowledged. First, this is a single-case observation, and the occult MCL component in the rectal specimen was recognized only retrospectively after systemic disease became clinically evident. Second, although multi-specimen targeted sequencing substantially increased the informativeness of the case, not all comparisons were fully platform-matched, and some specimens differed in panel design, analytic scope, and tissue composition; these factors limit direct cross-sample comparison and preclude robust phylogenetic inference. Exact tumor cellularity and assay-specific analytic thresholds were also not uniformly retrievable across all original reports. Third, as emphasized in the literature on synchronous or collision solid tumors and lymphomas, most reported cases remain largely descriptive, and the biologic relationship between coexisting components is often difficult to define with certainty (1). The contribution of the present report, therefore, is not to prove a common origin of rectal adenocarcinoma and MCL, but to document a diagnostically deceptive presentation in which retrospective pathologic review and component-resolved molecular analysis together clarified occult coexistence, partial molecular overlap, compartment-specific heterogeneity, and a clinically informative treatment course. More broadly, this case suggests that when systemic lymphoma emerges soon after treatment of colorectal carcinoma, especially in the presence of atypical lymphoid infiltrates in the original specimen, re-examination of prior pathology together with selective molecular reassessment may be highly informative.

## Patient perspective

The patient and her family initially understood the disease as rectal adenocarcinoma alone. The subsequent diagnosis of mantle cell lymphoma and retrospective reinterpretation of the surgical specimen substantially changed their understanding of the illness. After discussion of treatment options, they declined autologous transplantation and maintenance rituximab but accepted zanubrutinib maintenance. At the most recent follow-up, they expressed reassurance regarding the sustained remission and the clarified diagnosis. The reclassification from a single malignancy to a synchronous collision tumor helped explain the clinical sequence and supported shared decision-making about treatment intensity. At the most recent follow-up, they expressed reassurance about the sustained remission and the clarified diagnosis.

## Data Availability

The original contributions presented in the study are included in the article/**Supplementary Material**. Further inquiries can be directed to the corresponding author.
