# Supplementary material for: Case Report: Occult mantle cell lymphoma in a synchronous rectal collision tumor with adenocarcinoma: retrospective reclassification and multispecimen molecular profiling
Source: Front Oncol. 2026 Aug 20;16:1871711. doi: 10.3389/fonc.2026.1871711 (PMC13537932; doi:10.3389/fonc.2026.1871711)
Supplement: Supplementary file 1 [file SupplementaryFile1.docx]

**Supplementary Table S1. Variant-level extraction from the four original reports**

| **Specimen** | **Gene** | **Exon** | **cDNA change** | **Protein change** | **VAF (%)** | **Original report category** | **Comment** |
| --- | --- | --- | --- | --- | --- | --- | --- |
| **Specimen 1** | | | | | | | |
|  | TP53 | exon 7 | c.755_780dupTCACCATCATCACACTGGAAGACTCC | p.Gly262fs | 5.89 | Level 1 | Same exon 7 duplication later annotated as p.G262PfsTer92 / p.G262Pfs*92. |
|  | MYOM2 | exon 5 | c.558G>C | p.Gln186His | 58.64 | Level 3 | Also reported in Specimen 2; uncertain significance. |
|  | ARID1A | exon 18 | c.4993G>A | p.Gly1665Arg | 13.44 | Level 3 | Non-identical to the truncating ARID1A variant in Specimen 2. |
|  | KIR2DL1 | exon 7 | c.824C>T | p.Ala275Val | 17.01 | Level 3 |  |
|  | ATM | exon 63 | c.9156G>C | p.Trp3052Cys | 3.05 | Level 3 | Also reported in Specimen 2; uncertain significance. |
|  | IGLL5 | exon 1 | c.92T>A | p.Leu31Gln | 1.37 | Level 3 |  |
| **Specimen 2** | | | | | | | |
|  | NOTCH2 | exon 34 | c.7198C>T | p.Arg2400* | 41.35 | Level 1 | Dominant truncating alteration in the nodal lymphoma specimen. |
|  | ARID1A | exon 18 | c.4231C>T | p.Gln1411* | 43.72 | Level 2 | Truncating alteration; non-identical to the ARID1A variant in Specimen 1. |
|  | PCLO | exon 25 | c.15422C>T | p.Thr5141Met | 1.60 | Level 2 | Low-VAF Level 2 alteration; interpret cautiously. |
|  | MYOM2 | exon 5 | c.558G>C | p.Gln186His | 46.23 | Level 3 | Also reported in Specimen 1; uncertain significance. |
|  | ATM | exon 63 | c.9156G>C | p.Trp3052Cys | 54.99 | Level 3 | Also reported in Specimen 1; uncertain significance. |
| **Specimen 3** | | | | | | | |
|  | TP53 | exon 7 | c.755_780dup | p.G262PfsTer92 | 22.8 | IA | Same exon 7 duplication as in Specimens 1 and 4. |
|  | TNFRSF10B | exon 4 | c.476G>T | p.G159V | 55.31 | III | Reported as a variant of uncertain significance. |
|  | ZNF608 | exon 7 | c.4213G>A | p.V1405I | 51.4 | III | Reported as a variant of uncertain significance. |
| **Specimen 4** | | | | | | | |
|  | TP53 | exon 7 | c.755_780dup | p.G262Pfs*92 | 39.74 | Tier II | Same exon 7 duplication as in Specimens 1 and 3. |
|  | APC | exon 6 | c.642del | p.Q215Sfs*4 | 15.11 | Tier II | Canonical truncating APC alteration in the adenocarcinoma component. |
|  | RAD51D | exon 3 | c.196G>A | p.V66M | 41.50 | Tier III | Reported as a lower-priority alteration of uncertain significance. |

**Abbreviations and normalization notes**

- VAF, variant allele frequency; MSS, microsatellite stable; MMR, mismatch repair; CNV, copy-number variation.

- Original report categories were retained as reported because the four assays used different report-specific classification systems and are not directly comparable.

- For readability, panel names were summarized in Table 1. The original report label for Specimens 1 and 2 was “DLBCL hotspot screening (114 genes).”

- The TP53 exon 7 duplication was annotated differently across reports (p.Gly262fs, p.G262PfsTer92, and p.G262Pfs*92) but can reasonably be treated as the same exon 7 frameshift event.

- Specimen 1 should be interpreted as an archival composite bowel specimen later sequenced under hematology direction, not as a dedicated solid-tumor assay of a purified adenocarcinoma component.

- MYOM2 p.Gln186His was reported in Specimens 1 and 2 but remained a lower-priority alteration of uncertain significance and was therefore retained only in the supplementary extraction.

- ARID1A alterations in Specimens 1 and 2 were non-identical and should not be interpreted as a shared event.

- Absence of a given alteration in another specimen should be interpreted cautiously because panel content and analytic scope differed across assays.
